# Editorial: Targeting oxidative stress for protecting neurons against injury

**DOI:** 10.3389/fncel.2023.1327072

**Published:** 2023-11-06

**Authors:** Hulya Karatas

**Affiliations:** Institute of Neurological Sciences and Psychiatry, Hacettepe University, Ankara, Türkiye

**Keywords:** ischemic postconditioning, gliomas, febrile seizure, xenon treatment, intracerebral hemorrhage, NADPH oxidase 4, redox-related genes

Reactive oxygen species (ROS) are natural by-products of cellular metabolism. They play a role as secondary messengers in various neurological processes within the central nervous system (CNS). However, an imbalance between ROS production and removal leads to oxidative stress, which can harm genetic material, proteins, and cellular structures, and ultimately lead to cell death. Oxidative stress in the CNS has been associated with conditions like hypoxia, stroke, hypoglycemia, epilepsy, brain tumor formation, and several neurodegenerative diseases, including Alzheimer's, Parkinson's Disease, and Amyotrophic Lateral Sclerosis (Sharma et al., 2021; Cakir-Aktas et al., 2023). Consequently, researchers are exploring redox signaling pathways and molecules as potential targets for therapeutic interventions to mitigate neuronal damage. This collection of papers aims to enhance our understanding of the mechanisms involved in regulating antioxidant defenses and innovative strategies to reduce the generation of harmful oxidants in cases of neuronal injury. Within 4 original research articles published in this Research Topic, disease mechanisms were evaluated in in vivo and in vitro studies. Here I would like to summarize these papers. The publication by Esposito et al. focused on the potential benefits of ischemic postconditioning as a treatment for stroke recovery. Ischemic postconditioning, a technique involving controlled interruptions of blood flow, has shown promise in reducing brain damage after a stroke. However, its long-term effects and the underlying mechanisms are not well understood. This study aims to investigate how ischemic postconditioning promotes recovery after a stroke by experiments on rats subjected to a stroke-inducing procedure, with or without ischemic postconditioning. Various methods, including protein analysis and immunohistochemistry, were used to measure brain damage and assess the creation of new brain cells and blood vessels. An astrocyte inhibitor called fluorocitrate was used to investigate the role of astrocytes in postconditioning. The study found that the levels of Brain-Derived Neurotrophic Factor (BDNF) increased after postconditioning, especially in astrocytes. Inhibiting astrocytes using fluorocitrate prevented the protective effects of postconditioning and the growth of new blood vessels and brain cells. This is achieved through the release of BDNF and MMP9 by astrocytes. The research highlights the role of astrocytes and their interactions with other brain cells in promoting stroke recovery following postconditioning. This has potential implications for future stroke treatments, but further research is needed to explore other factors and long-term effects. Another study by Yuan et al. investigated the role of redox-related genes (ROGs) in high-grade gliomas (HGGs), a type of aggressive brain tumor. The research aims to understand how these genes affect tumor progression, patient prognosis, and the tumor microenvironment. HGGs are the most common malignant brain tumors in adults, and their treatment outcomes are poor. Redox balance, which involves reactive oxygen and nitrogen species (ROS/RNS) and antioxidants, plays a role in cancer progression, including HGGs. The study analyzed data from HGG patients in public databases (TCGA, CGGA) and established three cohorts: TCGA, CGGA, and West China Hospital. It identified 75 glioma-specific ROGs and categorized HGG patients into distinct groups based on their ROG expression patterns. Two main ROG expression clusters were identified: Cluster 1 had worse patient survival than Cluster 2. The study found associations between ROG patterns and clinical characteristics, including age, WHO grade, isocitrate dehydrogenase (IDH) mutation status, and more. The impact of ROGs on HGG aggressiveness differed between IDH mutant and wild-type tumors. Redox patterns were associated with immune profiles, such as immune cell infiltration and immune checkpoint expression. A glioma-redox risk score (GRORS) was established, serving as a prognostic tool for HGG patients. HGG patients with high GRORS had worse outcomes and exhibited immunosuppressive features in the tumor microenvironment. The study suggests that patients with high GRORS may benefit from immunotherapy. The research provides insights into the complex interplay between redox balance, immune response, and the aggressiveness of high-grade gliomas. In this Research Topic, a study by Cheng et al. focused on febrile seizures (FS) in children, which can lead to long-term neuronal damage and an increased risk of epilepsy. Currently, anti-epileptic drugs (AEDs) are used to treat FS, but they have limitations in preventing epilepsy and may harm brain development. The study evaluated the effectiveness of xenon gas in treating prolonged FS (PFS) and preventing epilepsy in young rats. Prolonged FS was induced in rat pups through hyperthermia. Pups in the xenon treatment group were exposed to a specific xenon-oxygen-nitrogen mixture for an hour after 90 min of PFS. The study measured various factors, including glutamate levels, mitochondrial oxidative stress, mitophagy (cellular process related to mitochondria), neuronal injury, seizures, and cognitive functions at different time points. Results showed that PFS in the neonatal period caused to spontaneous seizures and impaired learning and memory in the rat pups. PFS also increased levels of glutamate, mitochondrial oxidative stress, mitophagy, and neuronal injury. Xenon treatment reduced these negative effects caused by PFS and lowered the risk of PFS progressing into epilepsy. The study suggests that inhaling xenon gas could be a potential therapeutic approach to reduce neuronal injury and prevent the development of epilepsy in individuals who have experienced febrile seizures. This research highlights the potential benefits of xenon in the context of FS and its long-term consequences. The last study of this Research Topic is focused on intracerebral hemorrhage (ICH), a serious neurological condition associated with oxidative stress which is performed by Xie et al. This research investigated the role of a specific protein, NADPH oxidase 4 (NOX4), in the pathophysiology of ICH (Xie et al.). They used a rat model of ICH induced by injecting collagenase type IV. NOX4 expression was measured in the rat brains following ICH. NOX4 protein expression was knocked down by small interfering RNA. Various techniques, including western immunoblotting, immunohistochemistry, immunofluorescence, and enzyme-linked immunosorbent assay (ELISA), were used to assess the effects of NOX4 knockdown. Neurobiological scoring, brain water content measurement, and other assessments were conducted to evaluate brain injury and function. The study found that NOX4 expression increased in the brains of rats after ICH. NOX4 was mainly present in neurons, astrocytes, vascular endothelial cells, and microglia. When NOX4 was knocked down, oxidative stress in the brain significantly decreased, leading to improved neurobehavioral scores, reduced neuronal apoptosis, and enhanced blood-brain barrier function in rats with ICH. This study suggests that NOX4 expression is upregulated in response to ICH. This upregulation may contribute to an imbalance in oxidative stress in various brain cells, subsequently causing neuronal apoptosis and damage to the blood-brain barrier. The findings highlight the potential role of NOX4 in secondary brain injury following ICH, which proposes NOX4 as a target for future therapeutic interventions for ICH-related brain damage. In conclusion, this collection of papers explores the intricate mechanisms underlying oxidative stress and its impact on neurological conditions, offering valuable insights into potential therapeutic interventions. The findings highlight the promising avenues for further research and the potential for innovative strategies to mitigate the adverse effects of oxidative stress in a range of neurological disorders, ultimately advancing our understanding and treatment of these conditions.

## Author contributions

HK: Conceptualization, Writing—original draft.
